# Whole genome and exome sequencing of pancreatic neuroendocrine tumours to investigate PRRT response

**DOI:** 10.1530/ERC-26-0207

**Published:** 2026-08-06

**Authors:** Emma Boehm, Aidan Flynn, Alex Caneborg, Athena Hatzimihalis, Annette Hogg, Kelly Waldeck, Louise Jackett, Owen W J Prall, Catherine Mitchell, Grace Kong, Michael Michael, Tim Akhurst, Benjamin N J Thomson, William K Murray, Kwang Chin, Ben Lawrence, Cristin G Print, Joseph H A Vissers, Sean M Grimmond, Richard W Tothill, Rodney J Hicks

**Affiliations:** ^1^ Department of Clinical Pathology, The University of Melbourne, Parkville, Victoria, Australia; ^2^ Collaborative Centre for Genomic Cancer Medicine, The University of Melbourne, Parkville, Victoria, Australia; ^3^ Department of Cancer Imaging, Peter MacCallum Cancer Centre, Melbourne, Victoria, Australia; ^4^ Models of Cancer Translational Research Centre, Research Division, Peter MacCallum Cancer Centre, Melbourne, Victoria, Australia; ^5^ Sir Peter MacCallum Department of Oncology, University of Melbourne, Melbourne, Victoria, Australia; ^6^ Department of Anatomical Pathology, Peter MacCallum Cancer Centre, Melbourne, Victoria, Australia; ^7^ Department of Medical Oncology, Peter MacCallum Cancer Centre, Melbourne, Victoria, Australia; ^8^ Department of Surgical Oncology, Peter MacCallum Cancer Centre, Parkville, Victoria, Australia; ^9^ Department of Oncology, Faculty of Medicine and Health Sciences, University of Auckland, Auckland, New Zealand; ^10^ Maurice Wilkins Centre for Biodiscovery Hosted by The University of Auckland, Auckland, New Zealand; ^11^ Department of Molecular Medicine and Pathology, Faculty of Medical and Health Sciences, University of Auckland, Auckland, New Zealand; ^12^ Department of Medicine, The University of Melbourne, St Vincent’s Hospital, Melbourne, Victoria, Australia

**Keywords:** pancreatic neuroendocrine tumour, peptide receptor radionuclide therapy, genomics, DNA damage repair

## Abstract

Patients with pancreatic neuroendocrine tumours (PNETs) often have similar baseline clinical characteristics, including grade and molecular imaging phenotype, yet have highly variable responses to peptide receptor radionuclide therapy (PRRT). To identify genomic alterations and mutational patterns associated with PRRT treatment response and acquired somatic changes following PRRT exposure, whole genome or exome sequencing was applied to 40 PNET samples from 32 patients, including eight paired pre- or post-PRRT samples. The genomic profile of tumours reflected the known mutational landscape of PNET with *MEN1* (34%), *ATRX*/*DAXX* (47%) alterations and a recurrent pattern of aneuploidy (38%) detected. A recurrent *PSIP1*::*TBL1X* fusion of unknown function was also identified in four tumours. The disease control rate following PRRT using RECIST1.1 and molecular imaging criteria was 88% (28/32). No mutational features were found to be statistically associated with progression-free survival. There was no significant increase in tumour mutational burden in the post-PRRT tumours, nor recurrent emergent mutational changes in cancer driver genes to explain progression to higher-grade disease, when observed. However, a small indel signature (ID8) previously associated with DNA damage repair by non-homologous end joining (NHEJ) was higher in PRRT-exposed compared with PRRT-naive samples (23.8 vs 4.8%, respectively; *P* < 0.001). Thus, comprehensive DNA analysis of pancreatic NETs did not identify biomarkers predictive of PRRT response nor evidence for high-level PRRT-induced genomic instability or hypermutation, yet mutation signature analysis supports NHEJ as being important for DNA repair and survival of neuroendocrine cells following exposure to beta-particle radiation.

## Introduction

Peptide receptor radionuclide therapy (PRRT) with somatostatin receptor (SSTR)-targeting agents is a cornerstone of oncologic control in patients with advanced pancreatic neuroendocrine tumours (PNETs) (1). Despite having a molecular imaging phenotype ostensibly suitable for PRRT, patients can demonstrate divergent responses, with duration of disease control varying widely and approximately 15% of cases progressing through PRRT at first follow-up (2, 3, 4, 5).

Clinical and biological correlates of PRRT response have been described (6). High tumour grade and the related property of avidity on FDG PET/CT predict a shorter progression-free survival (PFS) (7, 8, 9). With respect to molecular biomarkers, the NETest®, a whole blood-based mRNA assay and its derived PRRT predictive quotient (PPQ) are reported to have predictive value for response to PRRT; however, the tumour genomic features underlying the whole blood RNA changes underpinning these tests are not known (10). The *MEN1/DAXX/ATRX* status has also been suggested to predict PRRT response in a small cohort of GEPNET (11). These findings need to be validated in larger patient cohorts.

PRRT involves delivery of a radioactive payload (e.g. ^177^Lutetium) through radiopharmaceutical ligand interaction with the somatostatin receptor. Energetic beta-particles released during ^177^Lutetium decay disrupt DNA mainly by causing single-stranded breaks (SSBs) either directly or indirectly through generation of reactive oxygen species; double-stranded DNA breaks also occur (12). Cellular mechanisms to recover from beta-particle exposure are complex and likely involve multiple DNA damage response (DDR) mechanisms (13). A subset of pancreatic NETs have inactivating variants of *BRCA2* and *CHEK2*, involved in the homologous recombination (HR) DNA repair pathway (14); however, *BRCA* alteration and HR deficiency have not correlated with better response to ^177^Lutetium-based radionuclide therapy in prostate adenocarcinoma (15). Preclinical studies have demonstrated that non-homologous end joining (NHEJ), an error-prone DNA repair mechanism, is a key process in cells treated with ^177^Lu-SSTR PRRT (16). Impairing NHEJ with DNA-PK inhibitors improves cell killing in ^177^Lutetium-resistant preclinical models, with the combination being explored in clinical trials (16, 17). Human data describing DDR in NET treated with PRRT have thus far been lacking.

PRRT has been implicated as a potential cause of de- or trans-differentiation of low-grade NET to NEC (18). However, there has, to date, been limited comparison of pre- and post-PRRT genomic features to support or understand this phenomenon, and the effect of temozolomide, which has been associated with hypermutation and microsatellite instability in tumours that acquire resistance, or underlying disease heterogeneity, has not been accounted for (18, 19, 20).

Here, a comprehensive molecular imaging and genomic analysis of a cohort of PNET patients treated with PRRT is presented in order to gain insights into the genomic determinants of treatment response and the consequences of PRRT exposure.

## Materials and methods

### Patients and study ethics

Adult patients with PNET treated with PRRT at the Peter MacCallum Cancer Centre (Peter Mac) who had tissue available for analysis, or who were planned to undergo re-biopsy or surgery following disease progression, were identified, and informed consent was obtained. This study was approved by the Peter Mac Human Research Ethics Committee (HREC LARF/114403/PCMM).

### Biospecimen acquisition and processing

The molecular imaging phenotype of patients recruited to the study was assessed by expert nuclear medicine physicians. Consultation with the interventional radiologist or surgeon involved ensured that lesions of interest, those with highest FDG-avidity (SUVmax) or differential growth rate, were selected for biopsy (if technically amenable) or, in the case of surgical resection, apportioned for genomic analysis. Where fresh tissue acquisition was not feasible, archival formalin-fixed paraffin-embedded (FFPE) blocks were obtained.

Tissue samples were assessed for tumour cell content by expert NET pathologists by haematoxylin and eosin staining of 4 μM cryosections. Ten serial 10 μM sections were taken for nucleic acid extraction adjacent to the H&E-reviewed sections. If required, microdissection of tissue was performed on ethanol-fixed and stained fresh tissue sections to dissect tumour regions from the sectioned material. Grade was reported using morphologic and mitotic thresholds as per WHO 2019 criteria (21).

### DNA and RNA sequencing

Whole genome sequencing (WGS), whole exome sequencing (WES) and whole transcriptome sequencing (WTS), where sufficient RNA was available, were performed using the DNA/RNA extracted from fresh or FFPE tissue. DNA was extracted from tissue samples and matched patient whole blood using the DNeasy® Blood & Tissue kit (Qiagen, Germany), and RNA was extracted from tissue using the miRNeasy mini kit (Qiagen, Germany) according to the manufacturer’s protocol.

*WGS:* DNA libraries were prepared using the Illumina TruSeq Nano (Illumina, USA) method with an input of 200 ng of DNA. Indexed libraries were pooled and sequenced to an average depth of 45× for the normal and 79× for tumour using paired 150 bp reads on the Illumina NovaSeq 6000 platform. Binary Base Call (BCL) files were demultiplexed and converted to FASTQ format using Illumina® *bcl2fastq* (version 2.20.0.422) with default settings. The *bcbio-nextgen* cancer somatic variant calling pipeline (version 1.2.4-76d5c4ba) (https://github.com/bcbio/bcbio-nextgen) was used to orchestrate alignment and quality control steps. Briefly, sequencing data in FASTQ format for tumour and matched normal were first processed by *Atropos* (version 1.1.28) (22) to clip homopolymers (minimum 8 bases) from the ends of reads. Trimmed data were then aligned to the human genome (GRCh38) with *BWA-mem* (version 0.7.17) (23) with predominantly default settings. The two exceptions were that the *-M* flag was enabled to mark shorter split read hits as a secondary alignment and seeds with >250 occurrences were skipped. Variant calling was performed with Sage (version 3.4.2), mutect2 (GATK version 4.6.0.0) and Strelka (version 2.9.10). Mutect calls were additionally filtered using the *LearnReadOrientationModel* and *FilterMutectCalls* modules. An intersection of variant calls from all three callers was performed using *bcftools* (version 1.20), and variants called by at least two callers were retained. The final variant list was annotated using *snpEff* (version 5.2c). Structural variants (SVs) were called with *GRIDSS* (version 2.13.2). *GRIDSS* output was annotated for repeat sequences using *RepeatMasker* (version 4.1.2.p1) and filtered for somatic SVs using *GRIPSS* (version 2.2). SVs were additionally annotated with *Linx* (version 1.21). Somatic copy number analysis was performed using *PURPLE* (version 3.6, https://github.com/hartwigmedical/hmftools) from the input generated by *GRIDSS, COBALT* (version 1.13) and *AMBER* (version 3.9). In patients with clinically reportable findings, variants were annotated using the Personal Cancer Genome Reporter (https://github.com/sigven/pcgr). The personal cancer genome report tool provides a statistical MSI classifier from somatic mutation profiles that separates MSI.H (MSI-high) from MSS (MS stable) tumours. Homologous recombination deficiency (HRD) was detected by HRDetect (24) and CHORD (25).

*WES:* WES was used for samples with a *GAPDH* multiplex PCR score of <4/8 (26). DNA libraries were prepared using SureSelect XT DNA kits (Agilent, USA), and exome regions were captured using the SureSelect Human All Exon V7 hybridisation and capture system. Indexed libraries were pooled and sequenced to an average depth of 157× for the normal and 75× for tumour using paired 150 bp reads on the Illumina NextSeq 1000, NextSeq 2000 or NovaSeq 6000 platform. Alignment from FASTQ and variant calling were performed as per WGS with the addition of a target region file for enrichment quality control. Somatic copy number analysis was performed using the *FACETS* (version 0.6.2) package for R.

*WTS*: Samples were prepared using the NEB-Next directional RNA-Seq kit (NEB, USA) and underwent 150 bp paired-end sequencing on the Illumina NovaSeq 6000 platform (Illumina, USA) according to the manufacturer’s instructions. WTS reads were aligned with *STAR* (version 2.7.11b) (27) using a two-pass approach. First, reads in FASTQ format were aligned using a *STAR* reference built with the GENCODE gene transcript annotation file (version 36, primary assembly annotation) and human reference genome with decoy HLA sequences (GRCh38). After alignment, the splice junction output from all samples was merged and supporting read evidence for each junction was summed. Junctions with less than 10 unique supporting reads were removed, and the resulting junctions were used to build a new *STAR* reference for realignment. Gene level counts were obtained from aligned data using *featureCounts* (version 2.0.6) (28). Fusion detection was performed using *Arriba* (version 2.4.0) (29). The effect of splice site variants detected by WGS was assessed via sashimi plots in the Integrated Genome Viewer (version 2.16.2).

### Definition of index PRRT for response assessment

See Supplemental methods (see section on Supplementary materials given at the end of the article) for a description of patient selection for PRRT and institutional protocol. Index PRRT was defined as the PRRT administered after the date of tissue collection. In cases where the biopsy was collected after PRRT with no subsequent cycles administered, response assessment was performed for most recent PRRT treatment block (see Supplemental Fig. 1 for a per-patient breakdown of the relationship between biopsy and PRRT).

### Response assessment

Response to PRRT was assessed at 3–6 months after completion of the index treatment block as per standard of care. Response and PFS were determined based on a composite of RECIST1.1 criteria and molecular imaging assessment using [68Ga]Ga-DOTA-octreotate (GaTate) and [18F]fluorodeoxyglucose (FDG) PET/CT (see Supplemental methods for definitions) (30, 31, 32, 33). PFS is reported from the date of the final cycle of PRRT received in the index treatment block. For the patients who contributed two biopsy specimens, PFS is reported in relation to the index PRRT for the earlier sample in order to control for repeated, unmeasured patient/biological factors contributing to PRRT response. Where relevant, participants were also given response labels taking into account the duration of PFS: *Exceptional responders* were defined as no structural or molecular evidence of disease despite extended follow-up (>5 years); *sensitive responders* defined as progression occurring 12 months or more after completion of PRRT; *sensitive non-durable responders* progressed less than 12 months after PRRT; and *resistant* patients progressed at first follow-up imaging.

### Research imaging analysis

Molecular imaging tumour volume (MITV) of pre-treatment and follow-up GaTate and FDG PET/CT studies were performed by a single reader using a semi-automated workflow created on MIM (MIM 6.9.4; MIM software, USA) with segmentation based on liver SUVmean +2 standard deviations (34) for both tracers. Manual removal of physiologic uptake and addition of pathologic regions, where relevant, was undertaken for each PET/CT study.

### Statistical analysis

Descriptive statistics are reported as counts and percentages. Non-parametric data are presented as median and range; Wilcoxon’s rank-sum and chi-squared tests are used to compare non-parametric data using the *wilcox.test* and *chisq.test* functions, respectively, from the *base* statistics package for R. Kruskal–Wallis testing used the *kruskal.test* function from the *base* statistics package for R. Dunn’s test using Holm’s method to correct for multiple testing for post hoc pairwise comparisons, using the *dunnTest* function from the *FSA* package for R, is used to compare non-parametric data among three or more groups. Spearman correlation was measured between continuous variables using the *cor* function from the *base* statistics package for R. Linear regression modelling was performed using the *lm* function from the *stats* package for R. Survival and hazards analyses, including Kaplan–Meir and Cox proportional hazards modelling, was performed using the *Survminer*, *survplot* and *finalfit* packages for R. We did not control for multiple testing due to the small, exploratory cohort. Figures were created using the *ggplot2*, *ComplexHeatmap*, *patchwork* and *swimplot* packages for R. All analyses were performed using R, version 4.2.2.

## Results

### A cohort of pancreatic NETs with variable response to PRRT

To investigate genomic features associated with PRRT response, 40 samples were collected from 32 patients with pancreatic NET (Table 1). Eight patients donated paired pre- and post-PRRT samples. Twenty-four samples were PRRT naive (median time from sample date to PRRT was 19 months, range: 0.75–128 months) and had not received other systemic oncologic therapies (Table 1, Supplemental Table 1). This included 18/24 unpaired samples and 6/8 paired samples. The median patient age at PNET diagnosis was 52 years (range: 15–72 years), and 20/32 (62.5%) patients were female. Eleven patients had functional tumours on biochemical testing. See Table 1 for patient and sample summary, Supplemental Fig. 1 for an overview of cohort clinical course and Supplemental Table 1 for a per-sample breakdown of treatments received.

**Table 1:** Patient demographics, details of index PRRT and sample details.

Patient demographics	
	*n* = 32 pts, 40 samples
Age (median, range)	52 (15–72)
Sex (F:M)	20:12
Hormones	
Non-functional	21
ACTH	2
ACTH/gastrin	1
Gastrin	2
Glucagon	3
Insulin	2
VIP	1
Lines of therapy prior to PRRT	
0	20
1*	12
Index PRRT details	
Activity administered (GBq; median, range)	29.3, 8.5–42.3 GBq
Number of cycles (*n*; median, range)	4, 1–5
Radiosensitising chemotherapy	
None	10
5FU	5
Capecitabine	9
CAPTEM	7
Etoposide	1

Sample details		
	Sample 1	Sample 2
Location		
Primary	12	0
Lymph node	5	1
Liver	15	5
Others	0	
Brain	-	1
Ovary	-	1
Grade		
1	4	0
2	15	2
3	12	5
NEC	0	1
Not specified	1	0
Systemic therapy exposure^#^		
SSA	1	2
Everolimus	1	0
5FU	1	0
Capecitabine	5	4
Temozolomide	4	2
Carboplatin/etoposide	1	0
XELOX	1	0
Pembrolizumab	1	0
PRRT	8	8
Unpaired samples	6	NA
Paired samples	2	8
No treatment	24	0
Unpaired samples	18	
Paired samples	6	

* Includes SSA, chemotherapy

^#^ Some have multiple exposures

Tumour grade of the earliest (or only) biopsy sample included G1 (*n* = 4), G2 (*n* = 15) and G3 (*n* = 12) with 1 sample being insufficient for grading. In the eight patients with serial samples, grade increased from G2 to G3 in half (*n* = 4) and G3 to NEC morphology in one case (discussed in detail below). The mean Ki-67 of the initial and second samples increased from 15 to 51% (*P* = 0.014, Welch’s two-sample *t*-test), reflecting the selection of progressive lesions for repeat biopsy.

**Figure 1:**
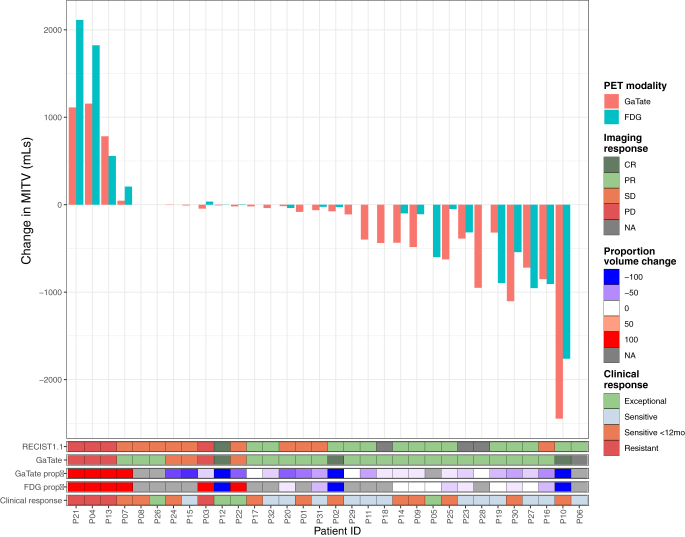
Molecular imaging-measured treatment response to index PRRT for baseline sample. The waterfall plot demonstrates the absolute change in molecular imaging tumour volume (MITV, mL) at the first follow-up imaging time point post-PRRT. The tiles represent the breakdown of imaging response per modality: RECIST1.1 is response on structural imaging; GaTate is response on GaTate molecular imaging criteria; GaTate prop^n^ is proportional change in MITV at follow-up compared with baseline; FDG prop^n^ is proportional change in MITV at follow-up compared with baseline. NA = not available for assessment.

Patients received a median of 29.3 GBq LuTate (range: 8.5–42.3 GBq) index PRRT over four cycles (range: 1–5). Twenty-two (68.8%) patients received radiosensitising chemotherapy with at least one cycle of index PRRT. On molecular imaging response criteria, three (9.4%) patients demonstrated a complete response (CR), 21 (65.6%) patients had a partial response (PR), three (9.4%) had stable disease (SD), four (12.5%) patients had progressive disease (PD), one patient did not have evaluable follow-up molecular imaging. The disease control rate was 88% (28/32) using molecular imaging response criteria and concordant with available RECIST1.1 results (Fig. 1). While the disease control rate was high at first follow-up imaging, the durability of control varied widely across the cohort: four patients had exceptional responses to PRRT with a PFS of >60 months (described in detail below); 13 patients had a PFS of 12–60 months; and 11 patients had responses lasting less than 12 months (Fig. 1).

### Genomic landscape of the advanced pancreatic NET cohort: expected and novel findings

We used a comprehensive sequencing approach to characterise the genomic landscape of pancreatic NETs treated with PRRT. WGS of fresh-frozen tissue (*n* = 12) or FFPE samples (*n* = 14) was used for 26/40 (65%) samples, and WES was used for poorer-quality DNA samples that failed a heuristic quality assessment threshold (*n* = 14).

### Expected PNET driver variants

**Figure 2:**
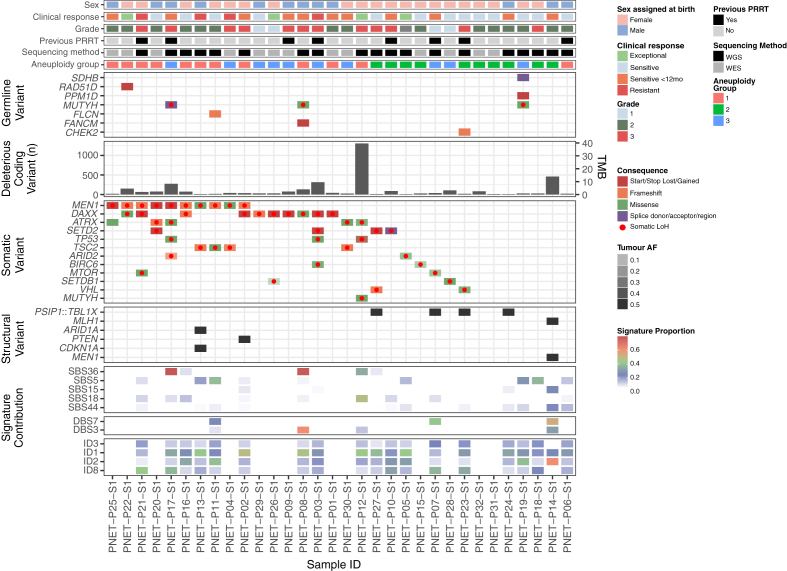
Clinical and genomic features of PNET patients treated with PRRT. Clinical response is defined as the duration of disease control prior to progression, *exceptional* is 60 months or longer; *sensitive* is 12–59 months; *sensitive <12 months* is 4–11 months; and resistant is 3 months or less (i.e. primary refractory cases). ‘Previous PRRT’ refers to whether the patient had radionuclide therapy before the sample was taken. ‘Aneuploidy group’ was determined using hierarchical clustering of copy number change as determined by copy number profile or Circos plot evaluation (see Fig. 3 for more details). The germline findings are annotated as to whether there was somatic loss of heterozygosity found in the sequenced tumour sample; denoted by a red circle. The COSMIC signatures are shown for samples that were sequenced using WGS (*n* = 19). Signature is displayed in terms of proportional contribution; denoted by colour. AF, allele fraction; PRRT, peptide receptor radionuclide therapy; TMB, tumour mutational burden (mutations/megabase); WES, whole exome sequencing; WGS, whole genome sequencing.

As in previous studies of metastatic PNET (14), the median tumour mutational burden (TMB) for this cohort was low at 1.12 mut/Mb. The median TMB of the PRRT-naive samples was 0.88 compared with 2.0 for the PRRT-exposed samples (*P* = 0.22, Wilcoxon’s rank-sum test). Across the cohort, patients had oncogenic or disrupting single nucleotide variants (SNVs) or indels in genes previously associated with pancreatic NETs, including *MEN1* (*n* = 10/32, 31.3%), *DAXX* (*n* = 10/32, 31.3%), *ATRX* (*n* = 5/32, 15.6%), *TSC2* (*n* = 4/32, 12.5%), *SETD2* (*n* = 4/32, 12.5%), *KMT2C* (3/32, 9.4%), *ARID2* (2/32, 6.2%), *VHL* (*n* = 2/32, 6.2%) and *MTOR* (*n* = 2/32, 6.2%). See Fig. 2 for recurrently altered genes and Supplemental Table 2 for variant details. Oncogenic *TP53* variants were found in samples from three patients with G3 disease, one of whom had NEC-like transformation of the second biopsy (described below). There were no pathogenic or likely pathogenic *MEN1*, *VHL* or *NF1* germline mutations observed in the cohort. Incidental findings of pathogenic or likely pathogenic germline variants were found in *FANCM* (14:g.45167133C>T), *FLCN* (17:g.17216394T>TG), *PPM1D* (17:g.60663388C>T) and *SDHB* (1:g.17053947C>A), not associated with PNET tumour syndromes, and without evidence of a second somatic lesion including loss of heterozygosity in the tumour sample to cause complete loss of tumour suppressor gene function.

Alterations in DNA damage repair genes were a feature of the cohort. Three patients had pathogenic germline *MUTYH* variants, and one case had a somatic *MUTYH* missense variant. In all four cases, there were concordant LoH and a dominant SNV-96 mutational signature SBS36 supporting *MUTYH* deficiency (35) (Fig. 2). The frequency of *MUTYH* variants in the PRRT-treated cohort was 12.5%.

A somatic *MLH1* SV in sample PNET-P14-S1 predicted to cause mismatch repair deficiency was also observed (Supplemental Table 3). PNET-P14-S1 had a high TMB at 13.9 mut/Mb and was called MSI-high using the PCGR method. Furthermore, PNET-P14-S1 had a high proportion of somatic variants that were assigned to SBS signatures 15 (21.6%) and 44 (19.0%), and doublet base substitution signature 7 (67.0%), each of which is associated with mismatch repair deficiency (35). This sample also had a gene-disrupting somatic *MEN1* SV. Patient PNET-P14 had a grade 2 PNET with a non-durable PR to 29.4 GBq LuTate (with radiosensitising capecitabine), progressing 6 months after PRRT. Given the limited treatment options, the patient received a further 23 GBq LuTate combined with capecitabine and temozolomide and progressed on the 2-month follow-up restaging imaging. A second biopsy (P14-S2, TMB 16.3 mut/Mb) was taken, which revealed progression to grade 3, with a Ki-67 of 90% and well-differentiated morphology. It is possible that the somatic *MLH1* variant and consequent mismatch repair deficiency conferred resistance to temozolomide in this case (19).

Other DNA damage repair gene alterations included germline *CHEK2* (patient P23, 22:g.28695238 TA>T) and *RAD51D* (patient P22, 17:g.35101206G>A) variants, but these were not associated with somatic LoH in the sequenced tumours. In addition, the sample from P22 analysed with WGS, PNET-P22-S2, was homologous recombination proficient based on CHORD and HRDetect classifiers (24, 25).

### Patterns of aneuploidy

**Figure 3:**
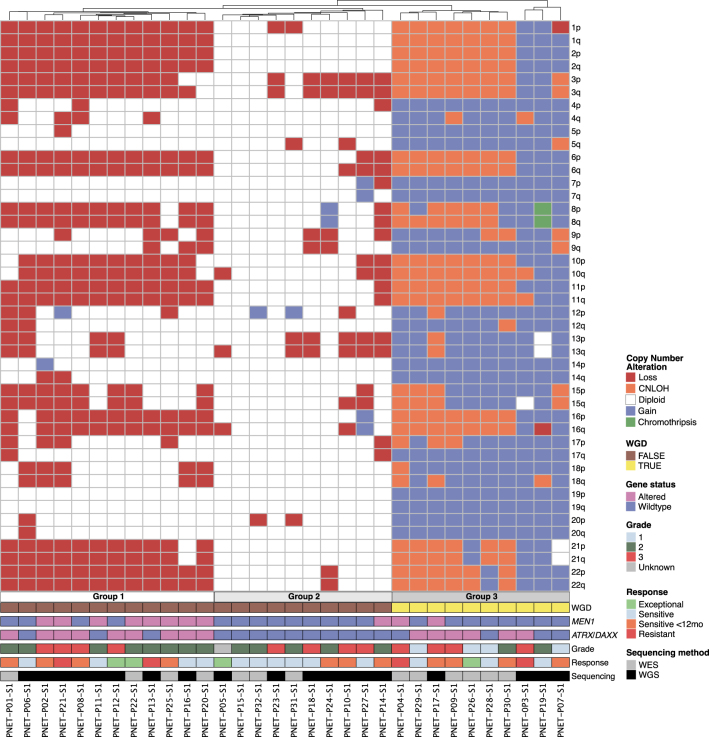
Copy alterations (baseline sample). Arm-level copy number alterations were determined based on the evaluation of output from *PURPLE* or *FACETS* and hierarchical clustering.

Characteristic patterns of somatic copy number alterations were identified in line with other previously published cohorts of pancreatic NET (14, 36, 37, 38). Hierarchical clustering of the initial samples from 32 patients identified three main groups (groups 1–3) (Fig. 3). Group 1 was characterised by a recurrent pattern of chromosomal losses at 1, 2, 3, 6, 8, 10, 11, 15, 16, 21 and 22. *MEN1* was altered in 8/12 and *ATRX*/*DAXX* was altered in 9/12 of group 1 cases. The mean ploidy of group 1 was 1.48. Group 2 contained 10 samples with predominantly diploid genomes (mean ploidy: 1.98) and a heterogeneous pattern of losses most often affecting chromosomes 3 (*n* = 6/10) and 13 (*n* = 5/10). Only one sample from group 2 had an *MEN1* alteration, and no samples had alterations of *ATRX*/*DAXX*. Group 3 consisted of 10 samples with whole genome duplication (WGD) events (mean ploidy: 3.49). Eight of these samples had copy number profiles consistent with initial losses of chromosomes 1, 2, 3, 6, 8, 10, 11, 15, 16, 21 and 22, followed by a subsequent WGD event (similar to the polyploid group described by Scarpa *et al.* (14)). As in prior studies (37), there were significant associations between the aneuploidy group and proportion of samples with *MEN1* alteration, *ATRX*/*DAXX* alteration and tumour grade (Fisher’s exact test; *P* = 0.016, <0.001 and 0.039, respectively). There was no statistically significant difference in median PFS among groups 1, 2 and 3 (15.5, 27.5 and 7 months, respectively, *P* = 0.86). Exceptional responders and resistant cases were found in each of the three groups.

### Novel *PSIP1* fusion

**Figure 4:**
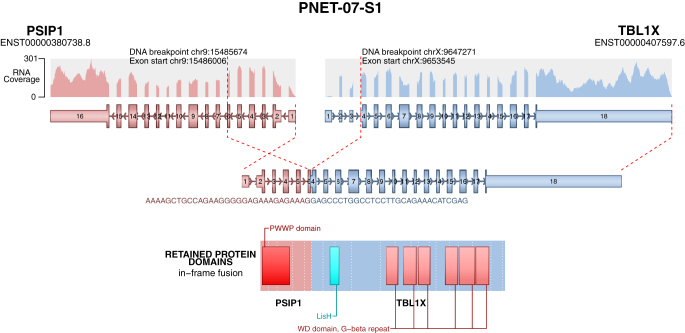
PNET-07-S1 PSIP1::TBL1X fusion RNA product. *PSIP1* intronic breakpoint between exons 6 and 7 and *TBL1X* intronic breakpoint between exons 3 and 4 with resultant fusion product (*Arriba* output). This is predicted to result in an in-frame fusion. The product retains the histone-binding PWWP domain of PSIP1 and the protein-binding WD repeat domain of TBL1X.

A novel recurrent *PSIP1::TBL1X* fusion was observed in samples from four individuals assessed by WGS: P24 with intronic breakpoints between *PSIP1* exons 7/8 (chr9: 15479049) and *TBL1X* exons 3/4 (chrX: 9649618, 5′ untranslated region, UTR), P07 with intronic breakpoints between *PSIP1* exons 6/7 (chr9: 15485674) and *TBL1X* exons 3/4 (chrX: 9647271, 5′ UTR), P23 with intronic breakpoints between *PSIP1* exons 6/7 (chr9: 15482240) and *TBL1X* exons 3/4 (chrX: 9650253, 5′ UTR) and P27 with intronic breakpoints between *PSIP1* exons 6/7 (chr9: 15484610) and *TBL1X* exons 3/4 (chrX: 9643465, 5′ UTR). RNA was available for two cases to demonstrate an in-frame fusion product for both cases (Fig. 4, Supplemental Table 4). All four tumours were functional (two insulinomas, one VIPoma and one ACTH-secreting tumour). *PSIP1* regulates transcription through interaction with menin (*MEN1*) and MLL1/2 (*KMT2A/D*) (39). In this cohort, the *PSIP1* fusion was mutually exclusive with *MEN1* alterations. Unlike the *MEN1*-altered samples, however, which were associated with recurrent patterns of chromosomal loss (aneuploidy group 1), the *PSIP1* fusion samples had few chromosomal alterations (group 2, *n* = 3) or few alterations and whole genome duplication (subset of group 3, *n* = 1). *PSIP1* has been proposed as an oncogene or tumour suppressor depending on cancer context (40, 41). *PSIP1* loss has been shown to impair homologous recombination and increase expression of DNA-PKc in cell line systems (42). The proportional contributions of SBS3, associated with homologous recombination deficiency, or the small indel signature ID8, as a surrogate for NHEJ activity, was not significantly increased in the *PSIP*-altered vs *PSIP*-intact tumours, noting small sample numbers and that this analysis does not control for PRRT exposure. The *PSIP1* fusion was not associated with differential PFS, with a median PFS of 16.5 months in the *PSIP1*-fusion cases compared with 19.5 months in the non-altered group (*P* = 0.41).

### Relationship between genomic features or grade and PRRT response

There was no association with *ATRX/DAXX* or *MEN1* mutational status and PFS. A Cox proportional hazards multivariate model was built (Table 2) containing clinical and molecular features, including grade, mean administered activity of LuTate for index PRRT, radiosensitising chemotherapy administration, TMB, *ATRX*/*DAXX* alteration and aneuploidy group as described above. WHO grade 3 disease was the only statistically significant predictor of progression (HR: 6.0, 95% CI: 1–32, *P* = 0.038) in this cohort.

**Table 2:** Cox proportional hazards model of clinical and genomic features predicting progression-free survival.

Progression-free survival: Cox proportional hazards model				
		All	HR (univariable)	HR (multivariable)
Grade	1	4 (12.9)	-	-
	2	15 (48.4)	2.54 (0.55–11.63, *P* = 0.230)	2.27 (0.42–12.27, *P* = 0.340)
	3	12 (38.7)	4.04 (0.87–18.65, *P* = 0.074)	6.00 (1.11–32.50, *P* = 0.038)
LuTate activity	Mean (SD)	27.2 (8.2)	0.99 (0.94–1.04, *P* = 0.715)	-
Radiosensitising chemotherapy	None	10 (31.2)	-	-
	Chemo given	22 (68.8)	0.47 (0.21–1.07, *P* = 0.072)	0.37 (0.13–1.06, *P* = 0.064)
Tumour mutational burden (Mut/Mb)	Mean (SD)	4.1 (8.9)	0.98 (0.93–1.03, *P* = 0.441)	-
Aneuploidy group	Group 1	12 (37.5)	-	-
	Group 2	10 (31.2)	0.92 (0.35–2.39, *P* = 0.860)	-
	Group 3	10 (31.2)	1.15 (0.44–3.00, *P* = 0.774)	-
*MEN1* status	Wild-type	21 (65.6)	-	-
	Altered	11 (34.4)	2.13 (0.94–4.80, *P* = 0.069)	1.46 (0.52–4.13, *P* = 0.472)
*ATRX*/*DAXX* status	Wild-type	17 (53.1)	-	-
	Altered	15 (46.9)	1.02 (0.46–2.26, *P* = 0.955)	-

### Exceptional responders and resistant cases

There were no clear recurrent genomic findings across the four exceptional responders to explain the favourable clinical behaviour (Table 3). Of note, P05 (female, Ki-67 not available, however, high FDG avidity at baseline suggests higher-grade disease) had a CR persisting 17 years after presentation with ectopic Cushing’s syndrome (the clinical course for this patient was previously described in (43)) and initial PRRT. PNET-P05-S1 had a largely diploid genome apart from chromothripsis events involving chromosomes 13 and 16 predicted to disrupt the tumour suppressor *LATS2*, as well as *CTCF*, which is critical for regulation of DNA topologically associating domain (TAD) boundaries (44). Interestingly, preclinical evidence has suggested an association between *CTCF* loss and radiosensitivity (45).

**Table 3:** Clinical and genomic features of exceptional responders and resistant cases.

Patient ID	Sample	Ki-67	Systemic Rx prior to index PRRT	Index PRRT regimen	Best PET response	PFS months	TMB mut/Mb	Driver (VAF)	Signature (proportional contribution)
Exceptional responders									
P05	PNET-P05-S1	NA (high FDG avidity)	Imatinib	7.4 GBq of InTide with 5FU + 30.1 GBq LuTate with 5FU*	CR	204	0.33	*ARID2* (32%)	ID2 (27%) SBS5 (27%)
P12	PNET-P12-S1	25%	NA	11.8 GBq LuTate with 5FU	CR	86	39.6 (MSS)	*MUTYH* (52%) *ATRX* (41%) *TP53* (41%)	ID1 (47%) SBS18 (45%) SBS36 (31%)
P22	PNET-P22-S1	5%	NA	26.7 GBq LuTate with capecitabine	SD	79	4.54	*MEN1* (84%) *DAXX* (89%)	NA (WES)
	PNET-P22-S2	6%	NA	26.7 GBq LuTate with capecitabine	SD	-	1.27	*MEN1* (56%) *DAXX* (41%)	ID1 (29%) SBS8 (28%)
P26	PNET-P26-S1	2%	NA	29.2 GBq LuTate with CAPTEM	CR	100	0.88	*DAXX* (51%) *SETDB1* (13%)	NA (WES)
Resistant cases									
P03	PNET-P03-S1	50%	FOLFIRI XELOX pembrolizumab	26.6 GBq LuTate with CAPTEM	PD	3	9.58	*TP53* (58%) *DAXX* (54%) *SETD2* (49%) *BIRC6* (44%)	ID9 (38%) SBS2 (38%) SBS13 (25%)
P04	PNET-P04-S1	40%	NA	15.8 GBq LuTate	PD	2	1.18	*MEN1* (52%) *TSC2* (44%)	NA (WES)
	PNET-P04-S2	60%	NA	NA	PD	-	1.39	*MEN1* (27%) *TSC2* (17%)	ID1 (34%)
P13	PNET-P13-S1	15%	NA	30.4 GBq LuTate with capecitabine	PD	3	0.39	*MEN1* (66%) *TSC2* (71%)	ID1 (39%)
	PNET-P13-S2	83%	NA	15.9 GBq LuTate with CAPTEM	PR	-	0.72	*MEN1* (48%) *TSC2* (42%)	ID1 (30%) SBS5 (23%)
P21	PNET-P21-S1	53%	LuTOC 27.5 GBq LuTate 20.2 GBq + capecitabine	8.5 GBq LuTate with CAPTEM	PD	3	2.00	*MEN1* (61%) *DAXX* (72%) *MTOR* (70%)	ID8 (38%) ID5 (27%) SBS40 (23%)

* Maintenance therapy was given: total of 33.2 GBq of LuTate over three years after initial treatment.

CAPTEM, capecitabine and temozolomide; FOLFIRI, folinic acid, fluorouracil, and irinotecan; LuTate, ^177^Lutetium-DOTATATE; LuTOC, ^177^Lutetium DOTATOC; XELOX, oxaliplatin and capecitabine.

Four cases demonstrated progression at first follow-up after index PRRT. Reflecting the results of the multivariate analysis, three of the four cases were WHO grade 3 at baseline biopsy. Three of the four cases had somatic alterations in the PI3K/AKT pathway, namely *TSC2* (*n* = 2) and *MTOR* (*n* = 1). Otherwise, there were no recurrent genomic features to distinguish the resistant group from the exceptional responders (Table 3), nor the rest of the cohort.

### ID8 signature contribution was higher in PRRT-exposed samples

To evaluate genomic changes in response to PRRT, the samples were grouped based on their relationship to any PRRT, being labelled as either ‘PRRT-naive’ or ‘PRRT-exposed’. This was done due to the paired sample group being small (*n* = 8, described in detail below) and pairs being analysed by different methodologies (WES/WGS).

**Figure 5:**
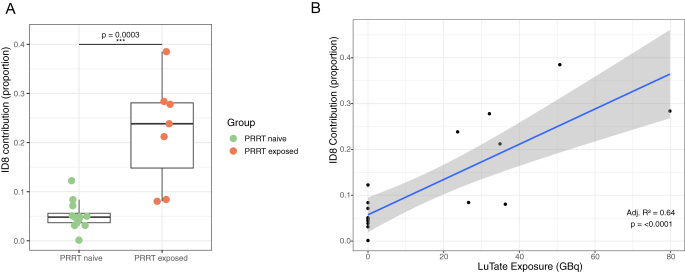
Detection of ID8 signature in tumours and associated with PRRT exposure. (A) Samples sequenced using WGS only are included. The baseline sample from each patient was categorised into PRRT-naive (*n* = 12) or PRRT-exposed (*n* = 7) groups, and the median proportion of the ID8 signature to total indels (signature contribution) was compared (Wilcoxon’s rank-sum test). (B) Relationship between activity of ^177^Lutetium DOTA-octreotate (LuTate) exposure and ID8 signature contribution. Displayed is the linear regression model line of best fit.

Signature analysis was only undertaken using the initial sample per individual that underwent WGS (*n* = 19/32). This was due to the inaccuracy inherent in comparing raw and proportional signature contributions of WGS (coding and non-coding regions) with WES (coding regions only) particularly in tumours with low TMB, such as our cohort. In the WGS cohort, the small indel signature ID8, associated with DNA repair by non-homologous end joining, was significantly higher in the PRRT-exposed group compared with the PRRT-naive group (median contribution: 23.8 vs 4.8%, respectively; *P* = 0.0003) (Fig. 5A). There was a moderately positive relationship between increasing ID8 contribution to total signature profile and increasing administered activity of LuTate (adjusted *R*^2^ = 0.64, *P* < 0.0001; Fig. 5B).

### Mutational and copy number features of paired samples

**Figure 6:**
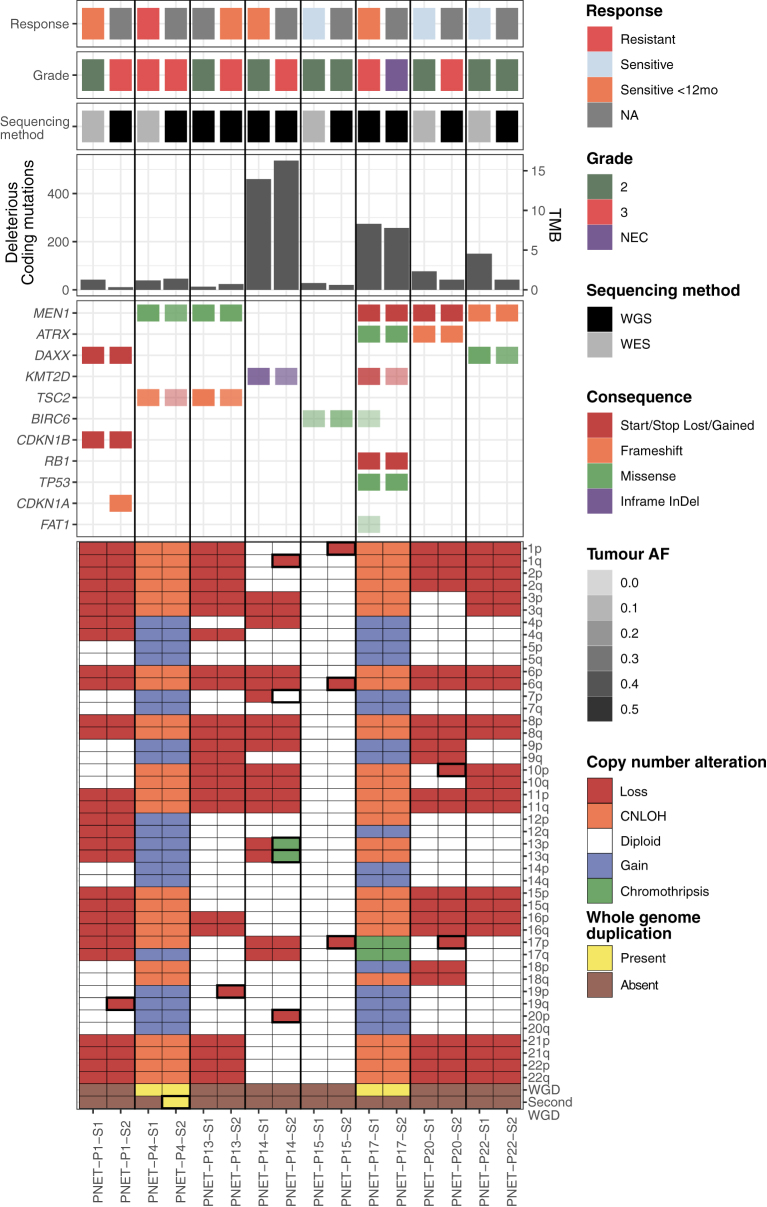
Clinical and genomic features of paired tumour samples pre- and post-PRRT. Eight patients had samples available before (S1) and after (S2) index PRRT event. Clinical response is NA if there was not an administration of PRRT after the biopsy was taken. Tumour mutational burden (TMB) and somatic variant profile is largely stable between S1 and S2. Copy number change in S2 is highlighted with black borders. Compared with the initial sample, PNET-P01-S2 demonstrated loss of 19q; PNET-P04-S2 had a second whole genome duplication event (with the baseline sample already demonstrating whole genome duplication following LoH of chromosomes 1, 2, 3, 6, 8, 10, 11, 15, 16, 17p, 18, 21, and 22); PNET-P13-S2 developed loss of 19p; PNET-P14-S2 developed loss of 1q, 20p, gain of 7p and chromothripsis at 13; PNET-P15-S2 lost 1p, 6q and 17p; and PNET-P20-S2 lost chromosome arms 10p and 17p. AF, allele fraction; NEC, neuroendocrine carcinoma; WES, whole exome sequencing; WGD, whole genome duplication; WGS, whole genome sequencing.

The increase in Ki-67 between the initial and second samples was not explained by emergence of single-gene variants classically associated with high-grade PNET (such as *TP53* or *RB1*), nor explained by loss of pre-existing *MEN1*, *ATRX* or *DAXX* variants (Fig. 6). There was no significant difference in median TMB between the first and second samples, 1.8 vs 1.27 mut/Mb, respectively (Wilcoxon’s test, *P* = 0.64). The second of the paired samples demonstrated additional chromosomal alteration compared to the initial sample in 6/8 cases (Fig. 6). There was no clear pattern of additional alteration seen.

### Evolution from NET to NEC-like morphology in a patient with extensive treatment exposure

**Figure 7:**
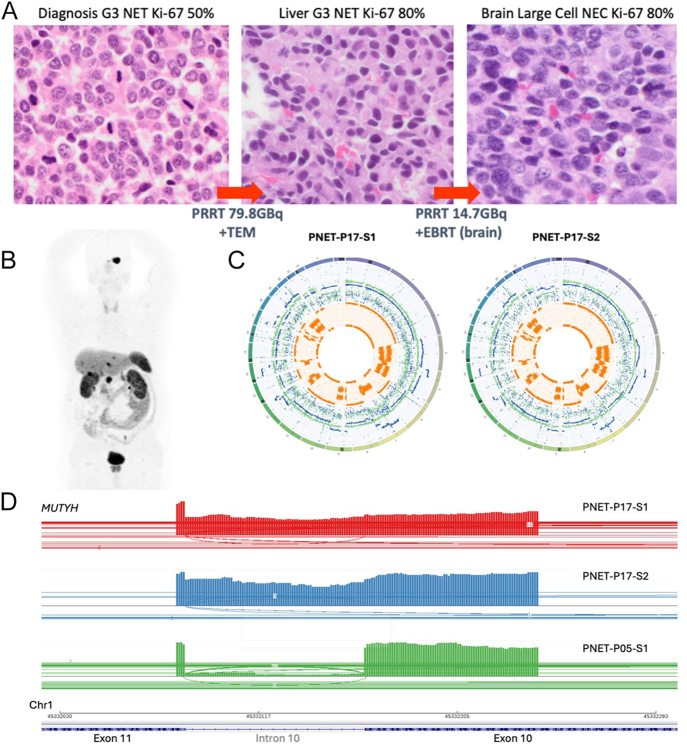
Histological, imaging and genomic features of case P17 with NET to NEC transformation. (A) Haematoxylin and eosin staining (400× magnification): *far left*: diagnostic liver biopsy (not sequenced) showing well-differentiated NET morphology with mitotic figures seen (the Ki-67 of this sample was 50%); *middle*: repeat liver biopsy (PNET-P17-S1) following 79.8 GBq PRRT demonstrating well-differentiated NET morphology and more frequent mitotic figures (the Ki-67 of this samples was 80%); *far right*: brain metastasis (PNET-P17-S2) resected following a further 14.7 GBq PRRT and external beam radiotherapy (EBRT), showing poorly differentiated, large-cell morphology and a Ki-67 of 80%. (B) GaTate PET/CT maximum intensity projection (MIP) prior to index PRRT (14.7 GBq, between S1 and S2) demonstrating intensely avid (Krenning 4, uptake greater than spleen) somatostatin receptor expression in the residual primary pancreatic lesion, small volume hepatic metastatic disease and a solitary Krenning 4 brain metastasis. (C) Circos plot (*PURPLE* output of WGS) of S1 and S2 demonstrating a minimal observable change in mutational pattern or copy number. RNA was available for both S1 and S2. (D) Sashimi plot (Integrative Genomics Viewer, IGV) at the MUTYH intronic splice site demonstrating abnormal splicing at the junction of exons 10 and 11 leading to intron retention compared with a non-affected control (PNET-P05-S1).

The samples taken from P17 demonstrate the clinical phenomenon of histological transformation from G3NET to a large-cell, poorly differentiated morphology consistent with NEC (Fig. 7A). The initial sample, obtained from a liver biopsy, was taken after 79.8 GBq of PRRT including three induction cycles with 5FU and two maintenance cycles with TEM. The second sample was obtained from a brain metastasectomy that retained somatostatin receptor expression on GaTate PET/CT (Fig. 7B) and had been exposed to an additional 14.3 GBq of PRRT (91.6 GBq total). The observed morphological evolution is not explained by acquisition of DNA alterations in known cancer genes (Fig. 6). The first and second samples both demonstrated the same clonal *MEN1*, *ATRX*, *TP53* and *RB1* variants at similar VAFs. Both samples from P17 had the characteristic SBS36 signature associated with *MUTYH* deficiency in the context of a germline splice-site variant with somatic LoH. The TMB did not significantly change between the first and second samples, 8.1 mut/Mb and 7.6 mut/Mb, respectively. The copy number profile of the two samples was similar, both belonging to polyploid group 3 (Fig. 7C).

## Discussion

Here, we performed comprehensive genomic analysis of advanced PNETs to understand the relationship between PNET DNA features and PRRT response. The genomic landscape of the cohort was consistent with that of pancreatic NET, with the additional finding of a novel recurrent *PSIP1*::*TBL1X* gene fusion. There were no recurrent statistically significant mutational features that predicted PRRT response. Importantly, there was no evidence that PRRT causes the emergence of mutation events associated with resistance nor triggers high-level genomic instability and progression, unlike what has been described in patients following DNA-alkylating chemotherapy exposure (e.g. temozolomide) (20). Despite PRRT not causing the acquisition of a large number of somatic changes, we found that the PRRT-exposed samples had an increased ID8 mutational signature, supporting the potential role for DNA repair by NHEJ in response to beta-particle radiation.

Comprehensive WGS and WES analysis revealed genomic features of PNET described in other landscape papers including recurrent somatic variants in *MEN1*, *TSC2* and *ATRX* or *DAXX* and predominantly low TMB (14, 37, 38). We did not find *CDKN2A/B* deleterious variants as in other metastatic cohorts (46); however, our study was limited by small numbers. Our cohort of metastatic PNETs demonstrate patterns of aneuploidy and whole genome doubling associated with poor prognosis and metastatic disease (37, 46, 47, 48). In addition, using WGS, we identified a *PSIP1*::*TBL1X* fusion in 4/32 (13%) individuals, supporting recent work that used optical genome mapping and long-read RNA-seq to identify a recurrent *PSIP1*::*TBL1X* fusion in 4/28 cases of metastatic PNET (49). Intriguingly, *PSIP1* encodes LEDGF/p75, which forms part of the COMPASS complex, linking menin (*MEN1*) and MLL1 (*KMT2A*) proteins to promote transcription (39). *PSIP1* frameshift and truncating variants have been found in small numbers of pulmonary NETs in tumours not otherwise harbouring *MEN1* alterations (50). The role of *PSIP1* may be tissue-specific as activation has been proposed as having oncogenic function in solid tumours (51), with high *PSIP1* expression associated with aggressive breast cancer subtypes and lower survival in one analysis of RNA expression data (40). Of further interest is the *TBL1X* fusion partner, which has a tissue-dependent function as part of the NCoR/SMRT nuclear receptor transcriptional corepressor complex or coactivator complex (52). *TBL1X* is currently known to regulate multiple hormone-medicated cellular functions through modulation of thyroid, oestrogen, androgen and PPAR receptor signalling (52). All four cases with *PSIP1*::*TBL1X* fusions were hormone secretory. Without functional studies, the activity and impact of this novel fusion in PNET are unknown.

In this highly selected cohort of metastatic PNET patients treated with PRRT, we found no statistical association of genomic features with progression-free survival. In prior studies, *MEN1* loss has been associated with increased overall survival and *ATRX*/*DAXX* (and alternative lengthening of telomeres) is associated with a higher likelihood of development of metastatic disease; however, the significance of these PNET-related gene alterations in relation to treatment outcome is less clear in the metastatic setting (46, 53, 54, 55, 56). Preclinical studies have demonstrated a role for *MEN1* and *ATRX* in DNA damage repair, including repair of radiation-induced DSBs (57, 58, 59). *ATRX/DAXX* knockout has been associated with radiosensitisation *in vitro* (58, 60, 61). We did not find a difference in PFS in patients receiving PRRT based on *MEN1* or *ATRX*/*DAXX* variant status. Conversely, a recent small (*n* = 28) retrospective clinical study of patients with GEPNET found an association between *MEN1*/*ATRX*/*DAXX* alteration and longer progression-free survival after PRRT (11). The *MEN1*/*ATRX*/*DAXX*-altered group, however, was pancreatic NETs, and outcomes were compared with patients who had small bowel and other NET subtypes, which have an intrinsically different radio-response and in which *MEN1*/*DAXX*/*ATRX* alterations do not typically occur.

Contrary to a prior study (18), we did not find strong evidence to support beta-particle radiation exposure causing a significant increase in mutation burden in PNET nor the acquisition of additional driver events that can explain tumour-grade progression. Despite the evolution from NET to a NEC-like morphology being clear in one of our cases (P17), the presumed driver *TP53* and *RB1* variants frequently associated with NEC (38) were pre-existing in this case and there was no emergence of other mutational features. Development of pancreatic NET from NEC is a contested issue, and in our case, the morphological changes could be explained by cellular response to ionising radiation rather than true transdifferentiation (62). Furthermore, we did not find a substantial increase in mutation burden or emergence of mutational signatures of genomic instability analysing the paired pre- and post-PRRT tumours. As expected with the selection of progressive lesions for repeat biopsy, we observed an increase in Ki-67 between paired samples. We did not observe late acquisition of variants, such as *CDKN2A* or *KRAS*, as described in a recent much larger cohort of paired primary and metastatic PNETS (46). We did observe additional arm-level or chromosome-level changes in the second sample in 6/8 pre- and post-treatment pairs, which may represent outgrowth of a pre-existing subclone with preferential survival fitness based on a combination of gene-dosage effects within regions of chromosomal changes (63). Multiregional sequencing or single-cell analysis of paired samples incorporating gene expression data is required to understand the evolution and biological impact of these events. Interestingly, despite PRRT not ostensibly causing resistance or grade progression through a distinct mutational mechanism, we detected the ID8 mutation signature following PRRT exposure, which is evidence to support prior preclinical findings (16, 17) that NHEJ-mediated DNA damage repair is an important mechanism in tumour cells responding to beta-particle DNA damage. These observations further support the rationale to target NHEJ through inhibition of DNA-PKc in combination with LuTate (16, 17) – an approach currently being explored in a phase 1 clinical trial (NCT04750954).

The limitations of our study include sample acquisition bias as some patients were selected for biopsy based on molecular imaging characteristics at the time of disease progression or because of particular response characteristics. Despite this, the cohort is reflective of the spectrum of disease response to PRRT seen in PNET in other studies (2). In addition, there was significant heterogeneity regarding prior treatment exposure, the cumulative administered activity in the index PRRT course and the use of radiosensitising chemotherapy, all of which confound evaluation of response and survival metrics. Nevertheless, this was controlled for in statistical analysis. The dose in Gy delivered to each lesion with index PRRT was not measured, and so this could not be controlled for in the analysis. The heterogeneity of lesional dose would be a critical difference to explain divergence of findings between *in vivo* and *in vitro* studies of genomic features and radiosensitivity.

## Conclusion

Comprehensive DNA analysis of pancreatic NETs treated with PRRT revealed an increased ID8 signature, supportive of a role for NHEJ in DDR following tissue exposure to PRRT, and a recurrent *PSIP1*::*TBL1X* fusion. Importantly, we did not identify a genomic ‘smoking gun’ for PRRT-induced hypermutation or genomic instability analogous to acquired mismatch repair deficiency and SBS11 in cases of temozolomide-induced hyper-progression (20). A multi-omics approach incorporating gene expression and epigenetic evaluation of larger numbers of samples pre- and post-PRRT integrated with accurate dosimetry is necessary to understand the genomic features of radio-response. Repeated tissue biopsies and tumour heterogeneity pose challenges to the field requiring innovative investigational approaches and application of novel genomic and molecular imaging technologies.

## Supplementary materials









## Declaration of interest

The authors declare that there is no conflict of interest that could be perceived as prejudicing the impartiality of the work reported.

## Funding

This project was funded by an Accelerator Award generously provided by the Neuroendocrine Tumor Research Foundation (NETRF).

## Ethical approval

This study was approved by the Peter MacCallum Cancer Centre Human Research Ethics Committee (HREC LARF/114403/PMCC).

## AI disclosure

No generative artificial intelligence (AI) tools were used in the preparation of the written or visual content of this manuscript.
