# Supplementary material for: Whole genome and exome sequencing of pancreatic neuroendocrine tumours to investigate PRRT response
Source: Endocr Relat Cancer. 2026 Aug 6;33(8):e260207. doi: 10.1530/ERC-26-0207 (PMC13452026; doi:10.1530/ERC-26-0207)
Supplement: Supplementary file 1 [file ERC-26-0207_supplementary_figure_1.pdf]

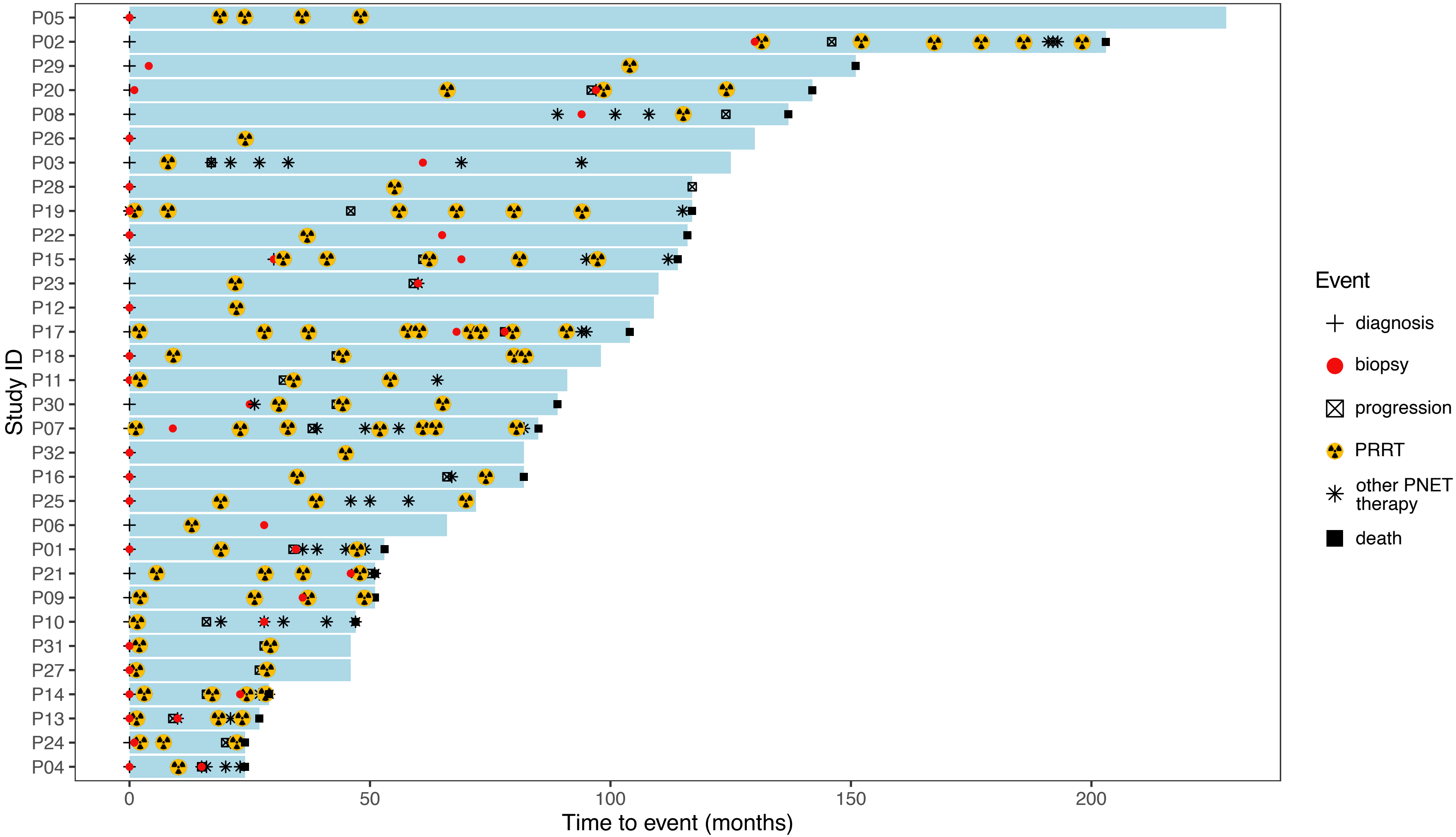

P05
P02
P29
P20
P08
P26
P03
P28
P19
P22
P15
P23
P12
P17
P18
P11
P30
P07
P32
P16
P25
P06
P01
P21
P09
P10
P31
P27
P14
P13
P24
P04
Study ID
0
50
100
150
200
Time to event (months)
Event
diagnosis
biopsy
progression
PRRT
other PNET therapy
death
