# Supplementary material for: Whole genome and exome sequencing of pancreatic neuroendocrine tumours to investigate PRRT response
Source: Endocr Relat Cancer. 2026 Aug 6;33(8):e260207. doi: 10.1530/ERC-26-0207 (PMC13452026; doi:10.1530/ERC-26-0207)
Supplement: Supplementary file 2 [file ERC-26-0207_supplementary_materials.pdf]

Whole genome and exome sequencing of pancreatic neuroendocrine tumours to investigate clinical and genomic response to peptide receptor radionuclide therapy

Supplemental methods

Patient selection for PRRT

Eligibility for PRRT included SSTR-tracer intensity greater than liver on tomographic imaging (i.e. modified Krenning score >2) at all visualised disease sites above the spatial resolution of the imaging technique used(1). All patients provided informed consent but were treated on compassionate grounds under the [Country name] Government Special Access Scheme (SAS), which allows the use of experimental therapies that have demonstrated efficacy in other studies to treat patients with life-threatening diseases. LuTate radiolabelling was performed under institutional protocol, with ^{177}Lu labelled to octreotate through chelation to a DOTA molecule.

During the study period, it was standard practice to consider radiosensitising chemotherapy in addition to PRRT for patients with histology or imaging suggestive of high-grade disease, and who did not have significant contraindication. When given, radiosensitising chemotherapy was added from PRRT cycle 2 onwards and considered for subsequent retreatment cycles. The regimen has changed over the study period. Early patients received 5-fluorouracil (5-FU, 200mg/m^2 daily, starting 2 days prior to PRRT for 2 weeks in total)(1). Subsequent institutional protocol included the use of oral capecitabine (825 mg/m^2 bd commencing 2 days prior to PRRT for 2 weeks) or CAPTEM (capecitabine 750 mg/m^2 oral bd D1-14 commencing 9 days prior to PRRT, with temozolomide 100 mg/m^2 bd D10-14 commencing on the day of PRRT for 5 days) (2).

Imaging response assessment

Molecular imaging response is reported as the response on PET/CT at first follow-up after index PRRT. FDG and SSTR PET/CT were repeated at approximately 3-6 months following completion of therapy and compared to baseline. The best observed molecular imaging response is also reported at any time point after PRRT. GaTate PET/CT response was defined as per criteria described in (3): *Complete SSTR Response:* Disappearance of all tracer-avid lesions (or) uptake indistinguishable or less than background physiological uptake if residual anatomical abnormality; *Partial SSTR Response*: Reduction in intensity of uptake by one modified Krenning score in at least one tumour site associated with decrease / stable size on CT (if measurable) or PET (if non-measurable on CT) (or) reduction in size of tracer-avid lesions (if measurable) regardless of intensity of uptake; *Stable SSTR Disease*: No partial response or progressive disease; *Progressive SSTR disease:* Development of new tracer-avid lesions (or) an increase in size of the tracer-avid lesions on CT (if measurable) or on PET (if non-measurable on CT) regardless of uptake.

Metabolic response was assessed on FDG PET/CT according to semiquantitative criteria (4). FDG lesion avidity was defined as SUVmax of a $1cm^3$ region of interest over the most avid part of disease; SUVmax <5 is “mild”, 5-10 is "moderate” and >10 is “intense” avidity(5).

Structural response was also evaluated for each case. Stable, partial response, complete response, or progression was defined by Response Evaluation Criteria in Solid Tumors criteria (RECIST1.1)(6). When available, contrast-enhanced CT images were directly compared. Otherwise, non-enhanced CT from PET (or SPECT) components of the study were assessed using scintigraphic uptake to identify the dominant lesions.

References

1. Kong G, Thompson M, Collins M, Herschtal A, Hofman MS, Johnston V, et al. Assessment of predictors of response and long-term survival of patients with neuroendocrine tumour treated with peptide receptor chemoradionuclide therapy (PRCRT). Eur J Nucl Med Mol Imaging. 2014;41(10):1831-44.
2. Kashyap R, Alipour R, Boehm E, Jewell K, RaviKumar AS, Cardin A, et al. Safety and efficacy of re-treatment with [(177)Lu]Lu-DOTA-Octreotate radionuclide therapy in progressive gastro-entero-pancreatic neuroendocrine tumours - a single centre experience. Eur J Nucl Med Mol Imaging. 2025.
3. Zidan L, Iravani A, Oleinikov K, Ben-Haim S, Gross DJ, Meirovitz A, et al. Efficacy and Safety of (177)Lu-DOTATATE in Lung Neuroendocrine Tumors: A Bicenter study. J Nucl Med. 2022;63(2):218-25.
4. Hicks RJ. The role of PET in monitoring therapy. Cancer Imaging. 2005;5(1):51-7.
5. Hofman MS, Hicks RJ. How We Read Oncologic FDG PET/CT. Cancer Imaging. 2016;16(1):35.
6. Eisenhauer EA, Therasse P, Bogaerts J, Schwartz LH, Sargent D, Ford R, et al. New response evaluation criteria in solid tumours: revised RECIST guideline (version 1.1). Eur J Cancer. 2009;45(2):228-47.
