# Supplementary material for: Whole genome and exome sequencing of pancreatic neuroendocrine tumours to investigate PRRT response
Source: Endocr Relat Cancer. 2026 Aug 6;33(8):e260207. doi: 10.1530/ERC-26-0207 (PMC13452026; doi:10.1530/ERC-26-0207)
Supplement: Supplementary file 3 [file ERC-26-0207_supplementary_table_1.pdf]

Supplemental Table 1: Systemic therapies received prior to biopsy.

	SSA	LuTate	JR11	LuTOC	Everolimus	Cap	Tem	5FU	Carbo /etop	FOLFIRI	XELOX	Pembro	EBRT
PNET-P01-S1	0	0	0	0	0	0	0	0	0	0	0	0	0
PNET-P01-S2	1	1	0	0	0	0	0	0	0	0	0	0	0
PNET-P02-S1	0	0	0	0	0	0	0	0	0	0	0	0	0
PNET-P03-S1	0	1	0	0	1	0	0	0	0	1	1	1	1
PNET-P04-S1	0	0	0	0	0	0	0	0	0	0	0	0	0
PNET-P04-S2	0	1	0	0	0	0	0	0	0	0	0	0	0
PNET-P05-S1	0	0	0	0	0	0	0	0	0	0	0	0	0
PNET-P06-S1	0	1	0	0	0	0	0	1	1	0	0	0	0
PNET-P07-S1	0	1	0	0	0	1	1	0	0	0	0	0	0
PNET-P08-S1	0	0	0	0	0	0	0	0	0	0	0	0	0
PNET-P09-S1	0	1	0	0	0	1	0	0	0	0	0	0	0
PNET-P10-S1	0	1	0	0	0	1	1	0	0	0	0	0	0
PNET-P11-S1	0	0	0	0	0	0	0	0	0	0	0	0	0
PNET-P12-S1	0	0	0	0	0	0	0	0	0	0	0	0	0
PNET-P13-S1	0	0	0	0	0	0	0	0	0	0	0	0	0
PNET-P13-S2	0	1	0	0	0	1	0	0	0	0	0	0	0
PNET-P14-S1	0	0	0	0	0	0	0	0	0	0	0	0	0
PNET-P14-S2	0	1	0	0	0	1	1	0	0	0	0	0	0
PNET-P15-S1	0	0	0	0	0	0	0	0	0	0	0	0	0
PNET-P15-S2	0	1	0	0	0	1	0	0	0	0	0	0	0
PNET-P16-S1	0	0	0	0	0	0	0	0	0	0	0	0	0
PNET-P17-S1	0	1	0	0	0	0	1	0	0	0	0	0	0
PNET-P17-S2	0	1	0	0	0	0	1	0	0	0	0	0	1
PNET-P18-S1	0	0	0	0	0	0	0	0	0	0	0	0	0

	SSA	LuTate	JR11	LuTOC	Everolimus	Cap	Tem	5FU	Carbo /etop	FOLFIRI	XELOX	Pembro	EBRT
PNET-P19-S1	0	0	0	0	0	0	0	0	0	0	0	0	0
PNET-P20-S1	0	0	0	0	0	0	0	0	0	0	0	0	0
PNET-P20-S2	0	0	1	0	0	0	0	0	0	0	0	0	0
PNET-P21-S1	1	1	0	1	0	1	0	0	0	0	0	0	0
PNET-P22-S1	0	0	0	0	0	0	0	0	0	0	0	0	0
PNET-P22-S2	1	1	0	0	0	1	0	0	0	0	0	0	0
PNET-P23-S1	0	1	0	0	0	1	1	0	0	0	0	0	0
PNET-P24-S1	0	0	0	0	0	0	0	0	0	0	0	0	0
PNET-P25-S1	0	0	0	0	0	0	0	0	0	0	0	0	0
PNET-P26-S1	0	0	0	0	0	0	0	0	0	0	0	0	0
PNET-P27-S1	0	0	0	0	0	0	0	0	0	0	0	0	0
PNET-P28-S1	0	0	0	0	0	0	0	0	0	0	0	0	0
PNET-P29-S1	0	0	0	0	0	0	0	0	0	0	0	0	0
PNET-P30-S1	0	0	0	0	0	0	0	0	0	0	0	0	0
PNET-P31-S1	0	0	0	0	0	0	0	0	0	0	0	0	0
PNET-P32-S1	0	0	0	0	0	0	0	0	0	0	0	0	0

1 = treatment received, 0 = not received.

5FU 5 Fluorouracil, Cap capecitabine, Carbo/etop carboplatin etoposide, EBRT external beam radiation therapy, FOLFIRI folinic acid fluorouracil irinotecan, JR11 ^{177}Lutetium- somatostatin receptor antagonist, LuTate ^{177}Lutetium-DOTA-tate, LuTOC ^{177}Lutetium DOTA-toc, Pembro pembrolizumab, Tem temozolomide, SSA somatostatin receptor analogue, XELOX oxaliplatin capecitabine
